# Influence of sintering temperatures on microstructure and electrochemical performances of LiNi_0.93_Co_0.04_Al_0.03_O_2_ cathode for high energy lithium ion batteries

**DOI:** 10.1038/s41598-022-13843-5

**Published:** 2022-06-10

**Authors:** Hye-Jin Park, Seong-Ju Sim, Bong-Soo Jin, Seung-Hwan Lee, Hyun-Soo Kim

**Affiliations:** 1 Next Generation Battery Research Center, Korea Electrotechnology Research Institute (KERI), Changwon, Republic of Korea; 2 Department of Materials Science and Engineering, Kangwon National University, Chuncheon, 24341 Republic of Korea

**Keywords:** Chemistry, Energy science and technology, Materials science

## Abstract

In this study, we present a method for synthesizing Ni-rich LiNi_0.93_Co_0.04_Al_0.03_O_2_ (NCA) with a high-energy cathode material by the solid-phase method. The sintering temperature plays a very important role in the electrochemical performance of the LiNi_0.93_Co_0.04_Al_0.03_O_2_ since it affects the crystallinity and structural stability. Therefore, various sintering temperatures (660 °C/690 °C/720 °C/750 °C/780 °C/810 °C) are studied to get optimum electrochemical performances. The electrochemical performance of LiNi_0.93_Co_0.04_Al_0.03_O_2_ sintered at 720 °C shows the highest discharge capacity of 217.48 mAh g^−1^ with excellent Coulombic efficiency of 87.84% at 0.1 C. Moreover, the LiNi_0.93_Co_0.04_Al_0.03_O_2_ sintered at 720 °C exhibits excellent rate-capability (181.1 mAh g^−1^ at 2.0 C) as well as superior cycle stability (95.4% after 80 cycles at 0.5 C). This is because optimized sintering temperature leads to good structural stability with low cation disorder and residual lithium content.

## Introduction

Recently, a rechargeable lithium-ion batteries (LIBs) have been widely used in various portable electronic devices and small mobile information technology (IT) devices. The high-capacity, energy density and power density are required for next-generation energy storage devices^1–4^. This is because, the application of LIBs has extended from small mobile IT devices to electric vehicles (EVs), hybrid electric vehicles (HEVs), and plug-in HEVs^5^.

LiCoO_2_ (LCO), the most common cathode material for LIBs, has been widely used because of its high coefficient of lithium ion diffusion, easy fabrication processing and high voltage^6,7^. However, LCO has major limitations such as low capacity, high cost, toxicity, poor rate capability and environmental pollution^8,9^. Especially, high energy and power density are essential towards in application of large-scaled devices such as EVs and HEVs. Therefore, many researchers have been focused on searching new cathode materials, which can have high power and energy density^10–13^. Among various candidates, LiNiO_2_ (LNO) cathode materials are being studied as an alternative to solve these problems such as high price and low capacitance of LCO. However, LNO, having low structural stability, is deformed from ‘layered structure’ to ‘spinel structure’ as the cycle progresses, finally resulting in slow lithium ion kinetics^14,15^.

To address these concerns, the LiNi_1−y−z_Co_y_Al_z_O_2_ (NCA) has been developed as a breakthrough^16^. It has also been reported that increasing the Al content provides higher capacity with stable performances^7,17–20^. The structural stability and the electrochemical performance of LNO can be improved by Co and Al substitutions for Ni site. Owing to smaller size of Co^3+^ (0.685 Å) and Al^3+^ (0.530 Å) than Ni^3+^ (0.740 Å), the substitution of Co and Al ions can lead to shrinkage of the a-axis, resulting in better stabilization of the layered structures which presents both high specific energy and power density.

Therefore, in this study, we synthesized NCA cathode material with various sintering temperatures (660–810 °C) to optimize the electrochemical performances. This is because crystallinity and structural stability, affected by sintering temperature, play an important role in the electrochemical performances of NCA^12,21–23^. Therefore, we investigated the effect of sintering temperature on structural stability and electrochemical performance of NCA.

## Experiment

For synthesis of NCA cathode material, Ni_0.93_Co_0.04_(OH)_2_ precursor (Fig. S1) was fabricated via a co-precipitation process. Ni_0.93_Co_0.04_(OH)_2_ precursor was mixed with, LiOH·H_2_O (Sigma-Aldrich) and Al(OH)_3_ (Sigma-Aldrich) as followed molar ratio (Li:Me:Al = 1.05:0.97:0.03). The mixture was calcined at 450 °C for 5 h and then calcined at different sintering temperature (660 °C/690 °C/720 °C/750 °C/780 °C/810 °C) for 15 h under an oxygen atmosphere.

For fabrication of cathode electrodes, NCA active materials, carbon black (Super P) and Polyvinylidene difluoride (PVDF) were mixed in the weight ratio of 96:2:2. N-methyl-2-pyrrolidone (NMP) solvent was then added to form slurry. The obtained slurry was casted on Al foil via bar coating process and then dried at 100 ℃ in vacuum oven for 10 h to remove the residual N-methyl-2-pyrrolidone solvent.

The 2032 coin cells were assembled with Li metal disc as anode in a glove box filled with argon gas, 1 M LiPF_6_ in ethylene carbonate, dimethyl carbonate and ethyl methyl carbonate (EC:DMC:EMC 1:1:1 v/v/v) as electrolyte and polyethylene was used as separator^11^.

Surface morphologies of the NCA particles were observed by FESEM (Field Emission Scanning Electron Microscopy, Hitachi S-4800, resolution limit 1 nm) at 1 K, 5 K and 15 K magnifications. The Ni, Co and Al element ratios were determined by using an EDS (Energy dispersive X-ray). Crystalline structure of the NCA powders was examined by XRD (X-ray diffraction, Philips, X-pert PRO MPD) analysis. The electrochemical performances were characterized via an electrochemical device (TOSCAT-3100, Toyo system).

## Results and discussion

**Figure 1:**
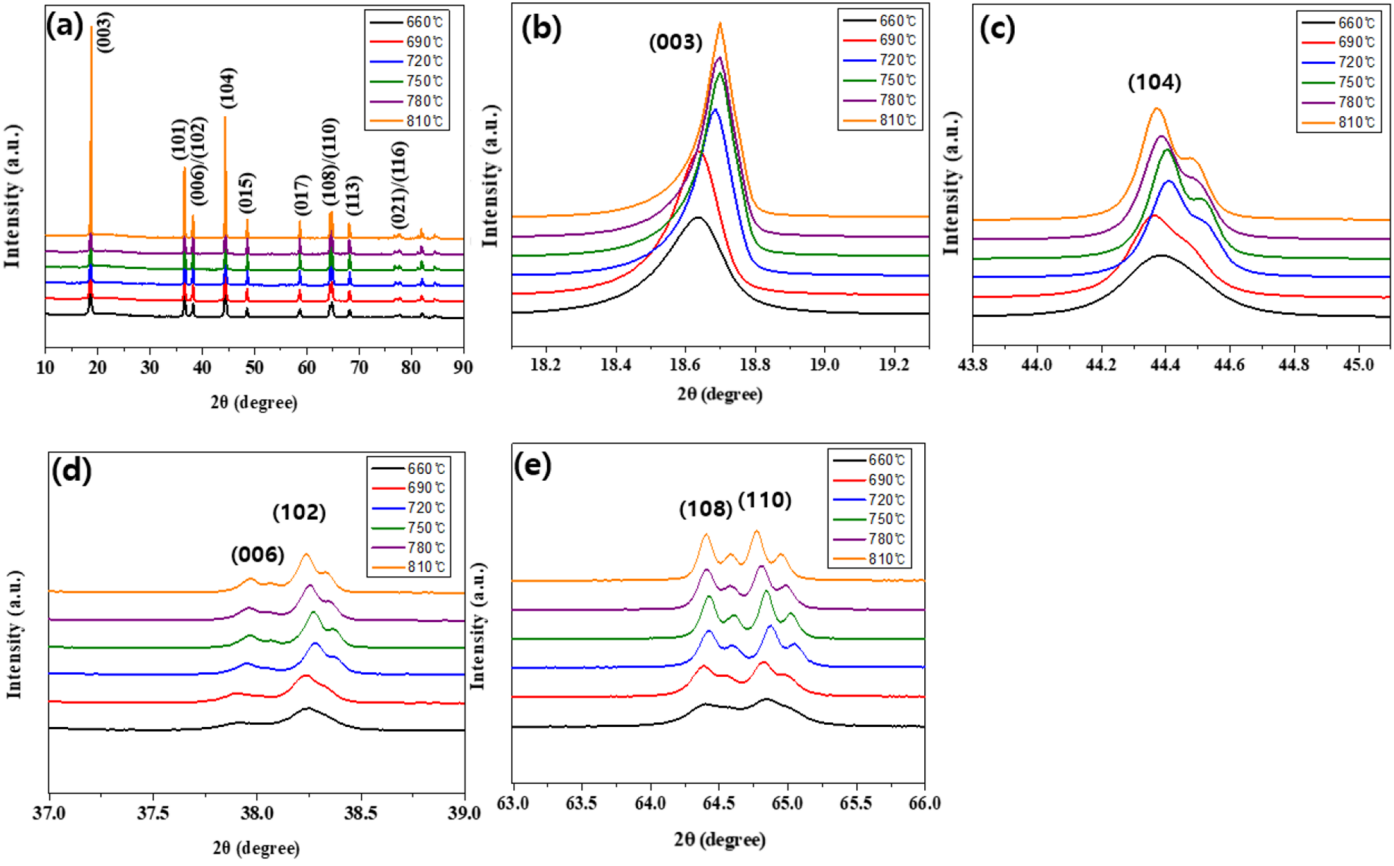
(**a**) XRD patterns and magnified views of the (**b**) (003) peaks, (**c**) (104) peaks, (**d**) (006)/(102) peaks and (**e**) (108)/(110) peaks.

**Figure 2:**
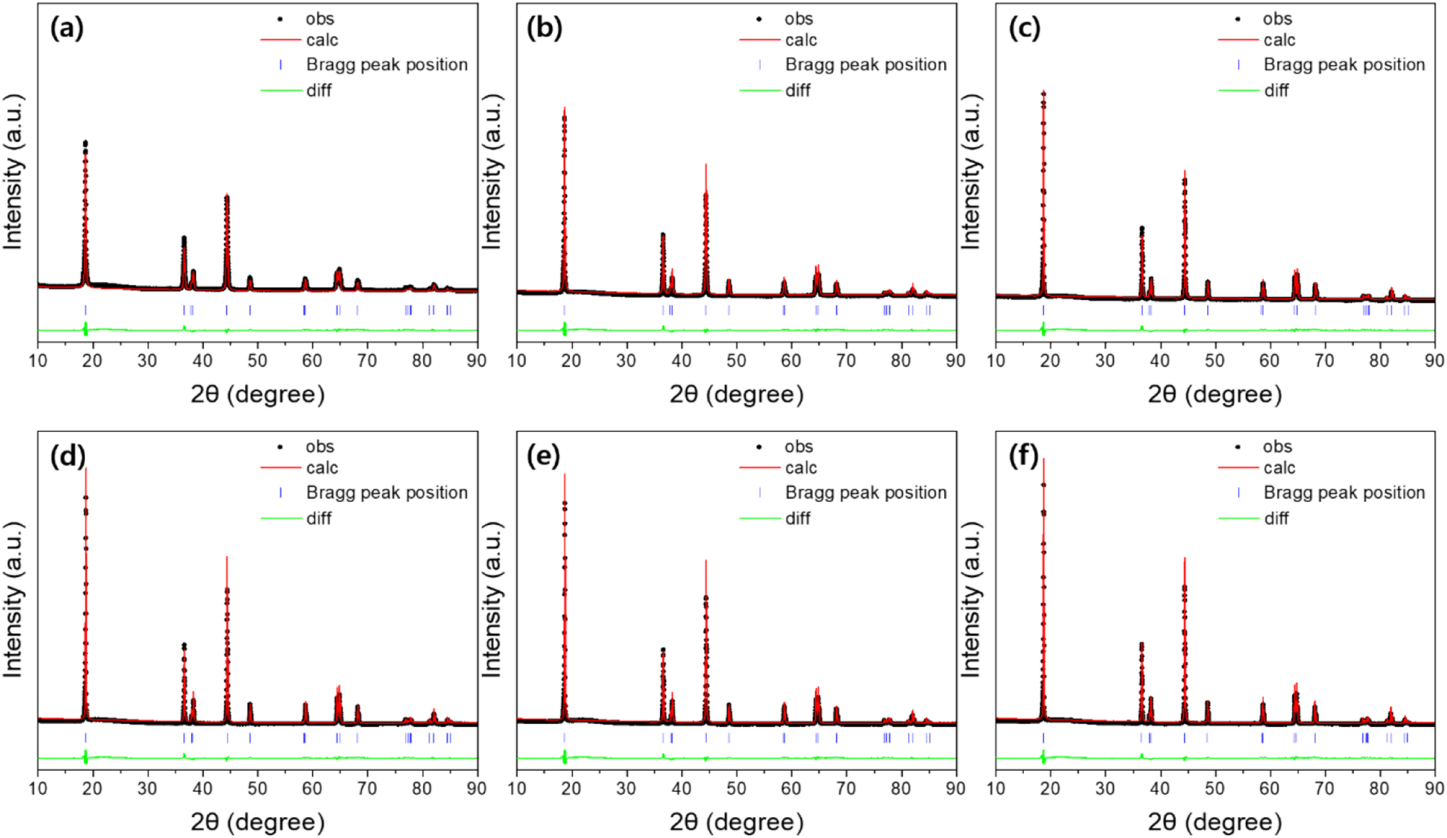
Reitveld refinement results of NCA sintered at (**a**) 660 °C, (**b**) 690 °C, (**c**) 720 °C, (**d**) 750 °C, (**e**) 780 °C and (**f**) 810 °C.

Figure 1 shows the (a) XRD patterns and magnified views of the (b) (003) peaks, (c) (104) peaks, (d) (006)/(102) peaks and (e) (108)/(110) peaks. All peaks correspond to the NCA with the α-NaFeO_2_ layered structure, belonging to hexagonal system (space group R-3 m). In addition, it was confirmed that there are no additional peaks, indicating a secondary phase, are not observed^24,25^. Rietveld refinement is measured to confirm the lattice parameters and I(003)/I(104) ratio, as shown in Fig. 2. All samples with an I(003)I(104) ratio greater than 1.2, has low cation disordering. In addition, the obvious splitting of I(006)/I(102) and I(108)/I(110) demonstrates that all samples except 660 °C are well-formed orderly layered structure. As shown in Table 1, the intensity ratio of I(003)/I(104) increased as the temperature increasing from 660 to 720 °C. However, it can be confirmed that the intensity ratio of the I(003)/I(104) is adversely decreased at the higher sintering temperature above 720 °C. It can be concluded that excessive sintering temperature deteriorates the structural stability of NCA.

**Table 1:** Structural parameters of NCA with various sintering temperatures.

	a (Å)	c (Å)	V (Å^3^)	c/a	I_(003)_/I_(104)_
660 °C	2.8687	14.1929	101.1524	4.9475	1.65
690 °C	2.8697	14.1920	101.2134	4.9455	1.72
720 °C	2.8692	14.1902	101.1686	4.9457	1.75
750 °C	2.8709	14.1891	101.2811	4.9424	1.66
780 °C	2.8723	14.1905	101.3903	4.9404	1.61
810 °C	2.8738	14.1881	101.4742	4.9371	1.54

**Figure 3:**
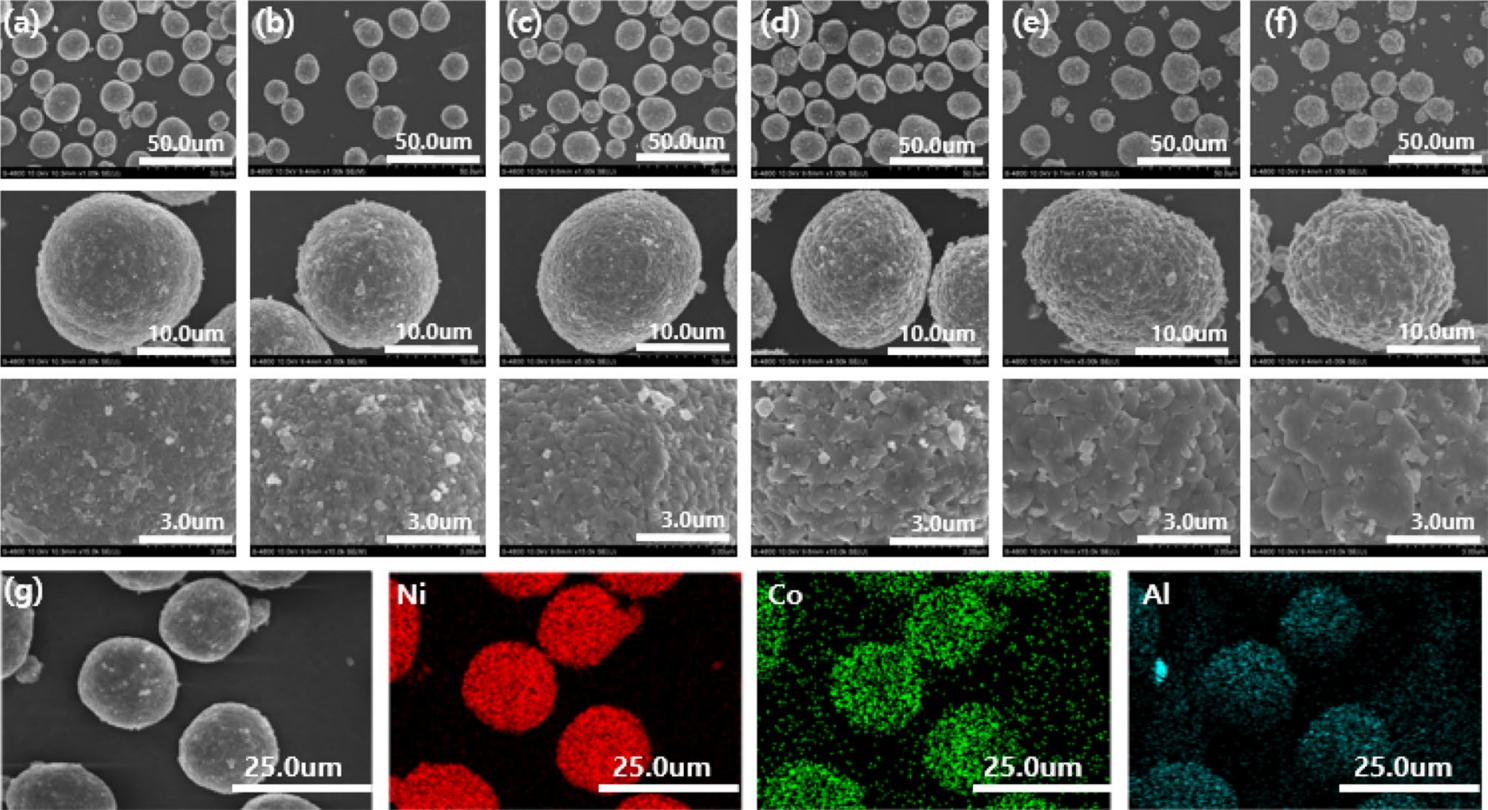
FE-SEM images of NCA sintered at (**a**) 660 °C, (**b**) 690 °C, (**c**) 720 °C, (**d**) 750 °C, (**e**) 780 °C and (**f**) 810 °C (**g**) EDS mapping of NCA sintered at 720 °C.

FE-SEM images of NCA sintered at different sintering temperatures [(a) 660 °C, (b) 690 °C, (c) 720 °C, (d) 750 °C, (e) 780 °C and (f) 810 °C] are shown in Fig. 3. From SEM images, all the samples show a spherical morphology with an average particle size of 10 ~ 20 um, which is composed of numerous primary particles (400 ~ 800 nm). Notably, the average size of primary particles increases as the sintering temperature increased from 660 to 810 °C. The average size of primary particle sintered at 660 °C is about 450 nm, much smaller than that of NCA (800 nm) sintered at 810 °C. Also, the clear Ni, Co, and Al peaks were observed in EDS mapping without any impurity peaks, as shown in Fig. 3g. It indicates that desired composition of NCA has been successfully synthesized.

**Figure 4:**
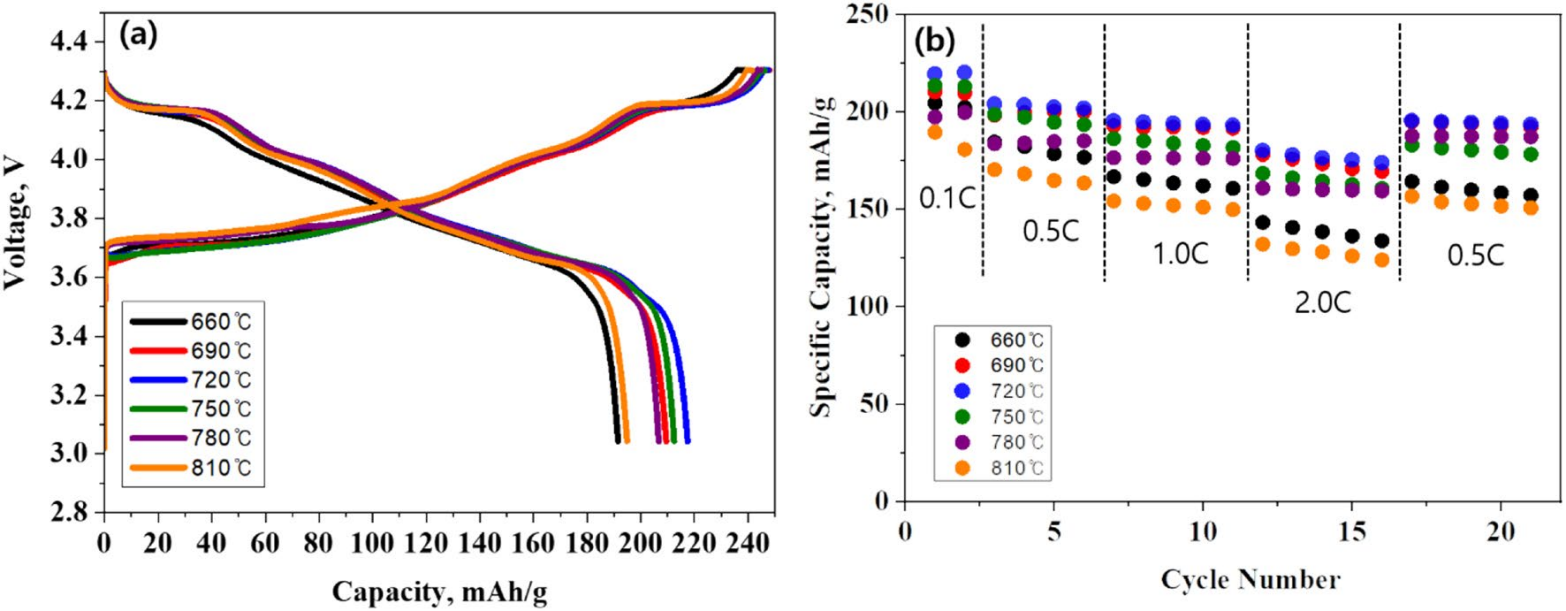
(**a**) Initial charge–discharge curves and (**b**) rate performances of NCA.

Figure 4a shows the initial charge–discharge curves of NCA with different sintering temperatures at current density of 0.5 A/g in the voltage range of 3.0–4.3 V. The voltage plateau around 4.2 V is due to the high Ni ion concentration in the NCA^26^. We can confirm that the capacity of all samples except 660 ℃ and 810 °C is not significantly different. Therefore, the sintering temperature does not significantly affect the initial charge–discharge capacities in the range from 690 to 780 °C. The sample sintered at 720 °C shows the highest discharge capacity of 217.48 mAh g^−1^ with excellent Coulombic efficiency of 87.84%. This is due to the low cation mixing and well-crystallized layered structure, as mentioned earlier (Fig. 1). Figure 4b presents the rate performance of NCA with different C-rates (0.1, 0.5, 1.0 and 2.0 C) in voltage range of 3.0–4.3 V. All the rate performances were measured at a fixed charge current density of 0.5 C while the discharge current density was measured at different current densities. The cell was cycled at each rate and then back to 0.5 C. The rate capabilities of all samples are decreased with increasing C-rate. However, it can be seen that the difference between the capacity values of samples increases, as the C-rate increases. Compared to the other cases, the NCA sintered at 720 °C maintains the highest capacities regardless of C-rates. More importantly, the NCA sintered at 720 °C still has the highest capacity of 195.3 mA h g^−1^ when the C-rate is decreased back to 0.5 C, indicating superior reversibility. This is because well-defined layered structure via optimized sintering temperature allows electrochemically active {010} planes of lithium ions and electron conduction despite the fast charge/discharge rate, resulting from high cation ordering and highly crystallized layered structure.

**Figure 5:**
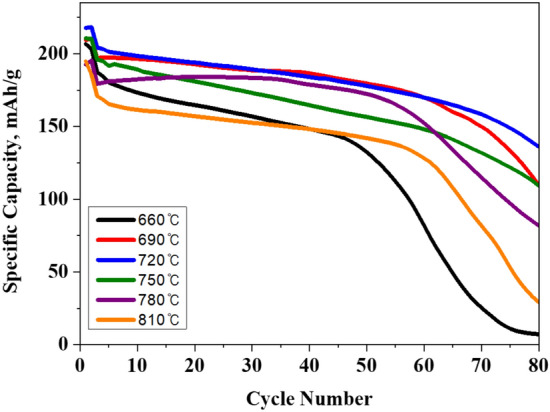
Cycling performances of NCA.

Figure 5 shows the cycle performances of NCA samples at 0.5 C in the voltage range 3.0–4.3 V. No obvious capacity fading is observed for all samples before 45 cycles. However, there is a difference in the rate of decrease for each sample after 45 cycles. Among the samples at different sintering temperatures, the NCA sintered at 720 °C possesses the highest capacity with capacity retention of 95.4% after 80 cycles. It can be explained by not only the rapid/durable electrochemical kinetics but also interfacial stability between electrode/electrolyte. On the other hand, the NCA sintered at 660 °C and 810 °C shows inferior cyclabilities compared to others. The samples sintered at 660 °C and 810 °C show a sharp decrease in capacity upon cycling. This is due to inferior crystallization (660 °C), disordered layered structure (660 °C and 810 °C), and longer Li ion-transfer channels (810 °C). Such drawbacks cause sudden drop in capacity during cycling since they could destroy the structural integrity of NCA, resulting in reduced reactivity of active material. It was reported that the hydrogen fluoride (HF), originated from reaction of water and LiPF_6_, is one of the most important factor for performance degradation. This is because HF elutes the transition metal ions in NCA, leading to deformation of layered structure. The optimum sintering temperature can suppress the negative effects on the NCA via boosting structural stability.

**Figure 6:**
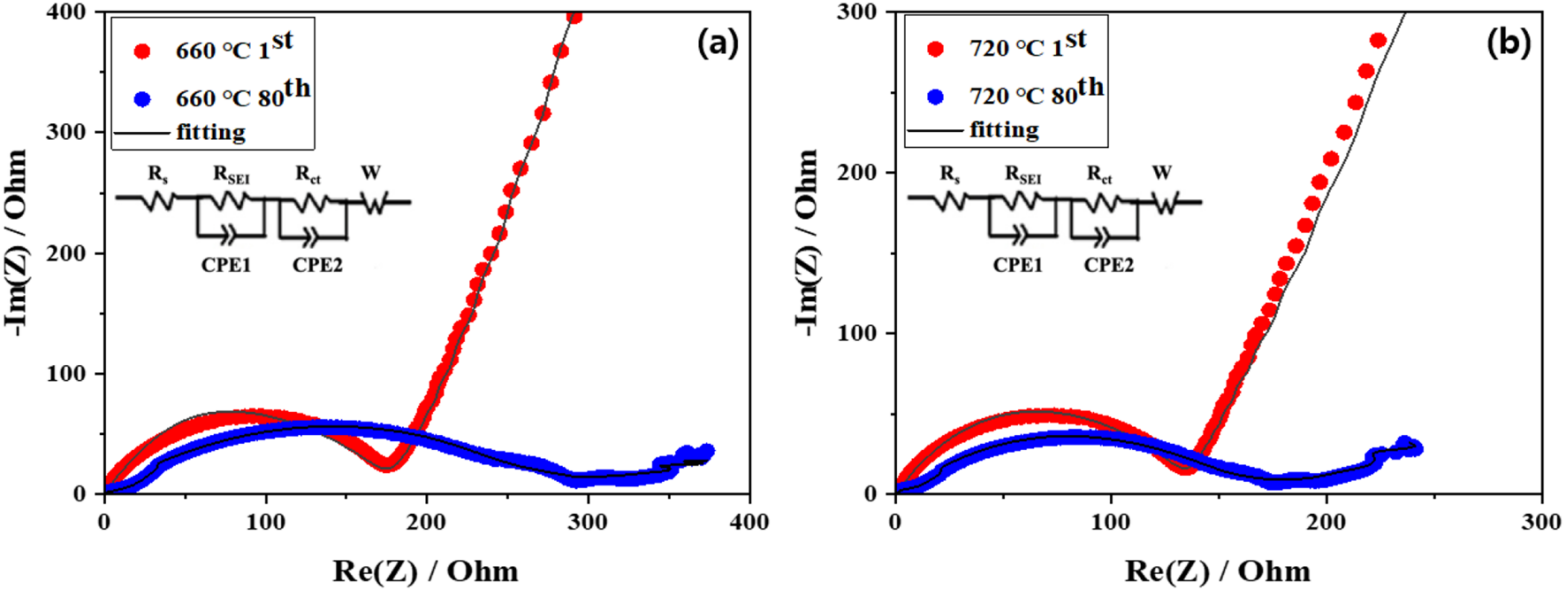
Nyquist plots of NCA sintered at (**a**) 660 °C and (**b**) 720 °C.

To better understand the electrochemical performances, EIS tests of NCA sintered at 660 °C and 720 °C are performed. Nyquist plots for the both samples after 1st and 80th cycles are shown in Fig. 6a and b. It is well known that Nyquist plots is composed of three components: electrolyte resistance (R_s_) in the high frequency, the charge transfer resistance (R_ct_) in medium frequency and the Warburg impedance in the low frequency^26–28^. Among three components, the R_ct_ can be regarded as a key parameter for the cathode impedance, affecting the electrochemical behavior. The R_s_ values of both samples are almost same since they use the same electrolyte. However, there is significantly difference in R_ct_ values between 1 and 80 cycles for both samples. Among them, the increase in R_ct_ value of NCA sintered at 660 °C (174.4 Ω to 295.9 Ω) is much larger compared to that of NCA sintered at 720 °C, as shown in Table 2. It can be inferred that highly crystallized layered NCA offers enlarged exposed active planes for Li ions. The R_ct_ value of NCA sintered at 720 °C shows about two-thirds than NCA sintered at 660 °C, resulting from enhanced charge-carrier transport at the NCA surface. Therefore, it can be concluded that optimized sintering temperature is beneficial to suppress the capacity fading during cycling based on structural stability of NCA.

**Table 2:** R_sf_ and R_ct_ values of NCA sintered at 660 °C and 720 °C.

	660 °C		720 °C	
	1 cycle	80 cycles	1 cycle	80 cycles
R_sf_ (Ω)	67.09	130.5	26.85	82.78
R_ct_ (Ω)	129.59	220.1	104.6	138.5

**Figure 7:**
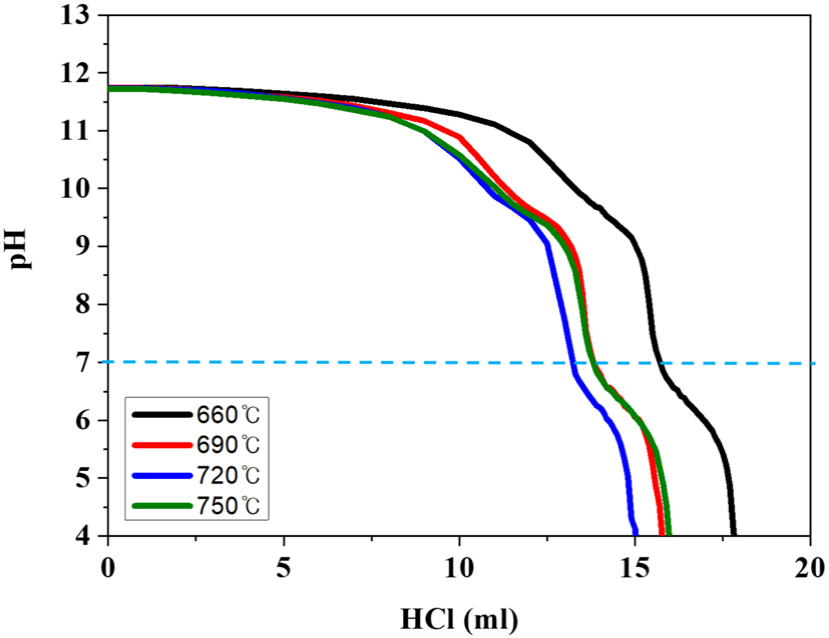
HCI-titration curves NCA with various sintering temperatures.

Figure 7 shows the HCl titration curves of NCA with different sintered samples. The residual lithium compounds (Li_2_CO_3_ and LiOH) is derived from (i) moisture absorption and (ii) spontaneous reduction of Ni^3+^ into Ni^2+^, accompanied by oxygen release. The amount of HCl used in the HCl titration up to pH 4 of NCA sintered at 660 °C is higher compared to that of 720 °C, as shown in Table 3. The amount of Li_2_CO_3_ and LiOH can be calculated via following equations^29,30^:

1 $${\text{LiOH }} + {\text{ HCl }} \to {\text{ LiCl }} + {\text{ H}}_{{2}} {\text{O}}$$

2 $${\text{Li}}_{{2}} {\text{CO}}_{{3}} + {\text{ HCl }} \to {\text{ 2LiCl }} + {\text{ H}}_{{2}} {\text{O }} + {\text{ CO}}_{{2}}$$

**Table 3:** Titration results of residual LiOH and Li_2_CO_3_ of NCA with various sintering temperatures.

	660 °C	690 °C	720 °C	750 °C
LiOH (wt%)	1.298	1.135	1.087	1.116
Li_2_CO_3_ (wt%)	0.635	0.576	0.546	0.635
Total (wt%)	1.93	1.71	1.63	1.75

The Li_2_CO_3_ is produced on the surface of LiOH by reaction with CO_2_, and then moisture remains, as follows:

3 $${\text{2LiOH }} + {\text{ CO}}_{{2}} \to {\text{ Li}}_{{2}} {\text{CO}}_{{3}} + {\text{ H}}_{{2}} {\text{O}}$$

Also, the CO_2_ and POF_3_ gases are generated by reaction with Li_2_CO_3_ and LiPF_6_. It can be represented by the following equation:

4 $${\text{LiPF}}_{{6}} + {\text{ Li}}_{{2}} {\text{CO}}_{{3}} \to {\text{ POF}}_{{3}} + {\text{ CO}}_{{2}} + {\text{ 3LiF}}$$

A portion of lithium is originated from the interior of NCA with the enhanced cation disordering. Therefore, the amount of residual lithium on the surface for sample sintered at 720 °C is small due to optimum sintering temperature^31^. It indicates that appropriate sintering temperature can effectively suppress the gassing and performance decay due to smooth surface chemistry without unwanted materials.

## Conclusion

In this study, we successfully synthesized the Ni-rich LiNi_0.93_Co_0.04_Al_0.03_O_2_ cathode materials with low Li^+^/Ni^2+^ disorder and high crystallinity under various sintering temperature conditions (660–810 °C). The effect of sintering temperature on the structural properties and electrochemical performances of NCA were investigated. The results indicate that the electrochemical performances of NCA are effected by sintering temperature. Among various sintering temperatures, NCA sintered at 720 °C shows the highest electrochemical performances based on excellent structural stability. It can be elucidated that NCA sintered at 720 °C has a good layered structure with high cation ordering thereby allowing fast and smooth Li ion and electron diffusion. Therefore, we can conclude that NCA sintered at 720 °C could be used as a promising cathode material for next-generation high-energy LIBs.

## Supplementary Information

Supplementary Figure S1.
