# Supplementary material for: Influence of sintering temperatures on microstructure and electrochemical performances of LiNi0.93Co0.04Al0.03O2 cathode for high energy lithium ion batteries
Source: Sci Rep. 2022 Jun 10;12:9617. doi: 10.1038/s41598-022-13843-5 (PMC9187639; doi:10.1038/s41598-022-13843-5)
Supplement: Supplementary file 1 — Supplementary Figure S1. [file 41598_2022_13843_MOESM1_ESM.docx]

*Supporting Information for*

Influence of sintering temperatures on microstructure and electrochemical performances of LiNi_0.93_Co_0.04_Al_0.03_O_2_ cathode for high energy lithium ion batteries

Hye-Jin Park^a^, Seong-Ju Sim^a^, Bong-Soo Jin^a^, Seung-Hwan Lee^b,*^ and Hyun-Soo Kim^a,*^

a Next Generation Battery Research Center, Korea Electrotechnology Research Institute (KERI), Changwon, Republic of Korea

b Department of Materials Science and Engineering, Kangwon National University, Chuncheon 24341, Republic of Korea

Corresponding Authors:

*E-mails: [shlee@kangwon.ac.kr](mailto:shlee@kangwon.ac.kr) and [hskim@keri.re.kr](mailto:hskim@keri.re.kr)


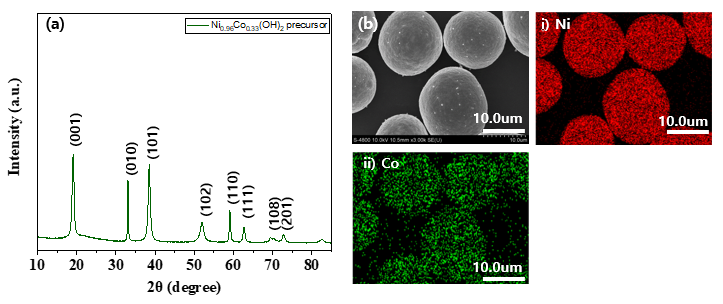


**Figure S1.** (a) XRD pattern and (b) SEM image and EDS mapping of precursor
